# Scavenging of lipid peroxyl radicals protects plasma lipids and proteins from peroxynitrite

**DOI:** 10.3892/br.2019.1253

**Published:** 2019-11-05

**Authors:** Ayman G Mustafa, Mahmoud A Alfaqih, Othman Al-Shboul, Ahmed Al-Dwairi

Biomed Rep 9:421-426, 2018; DOI: 10.3892/br.2018.1144

After the publication of the above paper, the first author, Ayman Mustafa, realized that his present address (Qatar University, Doha, Qatar) should have been included in the correspondence information for the manuscript. Therefore, the authors’ names and affiliations for this paper should have been presented as follows:

AYMAN G. MUSTAFA^1,3^, MAHMOUD A. ALFAQIH^2^, OTHMAN AL.SHBOUL^2^ and AHMED AL.DWAIRI^2^

Departments of ^1^Anatomy and ^2^Physiology and Biochemistry, School of Medicine, Jordan University of Science and Technology, Irbid 22110, Jordan

*Correspondence to*: Dr Mahmoud A. Alfaqih, Department of Physiology and Biochemistry, School of Medicine, Jordan University of Science and Technology, P.O. Box 3030 Ar Ramtha, Irbid 22110, Jordan

E-mail: maalfaqih@just.edu.jo

*Present address*: ^3^Department of Basic Medical Sciences, College of Medicine, Qatar University, Doha, Qatar

The authors regret this oversight in terms of their author affiliations, and apologize for any inconvenience caused.

